# Outcomes of Primary Percutaneous Coronary Intervention through a Transradial Approach in a Tertiary Care Cardiac Center

**DOI:** 10.7759/cureus.6484

**Published:** 2019-12-27

**Authors:** Najia A Soomro, Muhammad N Khan, Khalid Naseeb, Mahesh K Batra, Haris Majeed, Usman Bhatti, Tariq Ashraf, Musa Karim

**Affiliations:** 1 Cardiology, Liaquat National Hospital, Karachi, PAK; 2 Interventional Cardiology, National Institute of Cardiovascular Diseases (NICVD), Karachi, PAK; 3 Cardiology, National Institute of Cardiovascular Diseases (NICVD), Karachi, PAK; 4 Cardiology, National Institute of Cardiovascular Diseases (NICVD), karachi, PAK; 5 Statistician, National Institute of Cardiovascular Diseases (NICVD), Karachi, PAK

**Keywords:** primary percutaneous coronary intervention, forearm hematoma, stemi, transradial

## Abstract

Background

Transradial approach for percutaneous coronary intervention (PCI) is gaining popularity due to lesser bleeding and other access site related complications. This study aims to determine the in-hospital outcomes of primary PCI through a transradial approach in a tertiary care cardiac center.

Methods

Consecutive patients with ST-segment elevation myocardial infarction presenting within 12 hours with symptoms without a previous history of thrombolytic therapy, coronary angioplasty, or cardiac surgery were included in the study. All patients underwent a diagnostic angiogram followed by primary PCI of the infarct-related artery through a radial route and were kept under observation during the hospital stay for forearm hematoma or mortality.

Results

A total of 180 patients were included in this study, with a mean age of 52.04±7.31 years. Majority (87.2%) of the patients were male, and diabetes (72.8%) was the most commonly observed co-morbid condition followed by hypertension (67.2%). Hospital mortality rate was 3.9% (7 patients), and post-procedure forearm hematoma was noted in 5.6% (10 patients). An increased mortality rate was found to be associated with age above 50 years (7.1% vs. 0.0%; p=0.012) and non-hypertension (8.5% vs. 1.7%; p=0.026). An increased incidence of forearm hematoma was found to be associated with age above 50 years (10.2% vs. 0.0%; p=0.002), diabetic mellitus (7.6% vs. 0.0%; p=0.047), hyperlipidemia (11% vs. 0.0%; p=0.001), and non-smoking (10.2% vs. 0.0%; p=0.003).

Conclusion

Our study showed that primary PCI through a transradial approach is a safe option with excellent success rates in terms of both mortality rates and morbidity such as forearm hematoma.

## Introduction

The primary goal in the treatment of ST-elevation myocardial infarction (STEMI) is to restore perfusion of the myocardium through the recanalization of the vessel that is blocked. Reperfusion in the early stages has shown to produce better results [1-2]. For reperfusion, there are various strategies that can be applied, but the most common ones are thrombolytic therapy and primary percutaneous coronary intervention (PPCI) [3-4]. Various studies across different populations have proven that in relation to mortality, stroke, and reinfarction, PPCI is a more successful and efficient method of treatment in comparison to thrombolysis in the treatment of STEMI [5].

In combination with result-oriented pharmacological therapies, the use of PPCI in relevant subjects decreases both mortality and morbidity rates across a variety of sub-groups with varying risk levels, bringing major benefits to the field of medicine [1,6-9]. With the continual developments and evolutions in both device technology and antithrombotic therapy, PCI is the treatment of choice for a greater number of patients across the world with a wider range of lesion complexities [8,10]. PPCI can be performed through the transfemoral or transradial routes. Transradial approach is now gaining popularity due to fewer bleeding complications associated with it however there is a definite learning curve to acquire transradial skills [11-12].

In spite of an extended history, transfemoral approach was preferred over transradial approach by cardiologists, and its application has more than often been confined to a very small niche procedure due to the fact that physicians lack required training, experience, and exposure to this technique coupled with limitations of the available technology related to the technique [8,13-14]. Up until very recent years, a very limited number of studies have been carried out to evaluate the history behind the transradial method and very limited literature was available advocating its use in day-to-day PCI procedures [15]. Now there has been growing evidence suggesting that the use of the transradial method instead of the transfemoral method for PCI and that it is linked with relatively substantial decreases in post-procedure bleeding problems [16-19]. Rates of procedural failures have decreased with the increase in the experience of using the transradial method. A study found substantial reductions in failure to access, insertion time of the sheath, and cumulative time of the procedures to be correlated with an annual volume of transradial cases [20].

Transradial approach for PPCI is gaining rapid acceptability among the cardiologists in our part of the world; however, there is a definite learning curve and data is lacking regarding the safety of transradial approach for PPCI of patients with STEMI in our population. This lack of studies in the field, therefore, provides a perfect platform and rationale to perform a study that could help in determining the rates of death and complications related to PPCI through a transradial approach.

## Materials and methods

A total of 180 consecutive adult patients presented to the emergency room within 12 hours of the onset of chest pain were included in this study. We included patients of both genders, aged above 18 years of age, and diagnosed with STEMI. Patients with pre-existing stable coronary artery diseases or a history of coronary angioplasty or coronary artery bypass surgery were excluded. Also, patients who had cardiogenic shock, non-palpable radial artery, puncture site infection, and potential for future arteriovenous fistula surgery were excluded from the study. STEMI was defined as typical chest pain (for at least 20 minutes) and electrocardiographic evidence of ST-segment elevation of >1 mm in two contiguous leads or new-onset left bundle branch block. All the patients selected for the study had to undergo a diagnostic angiogram, which was followed by the PPCI of the culprit artery with or without stenting. A signed informed consent form was obtained from all the patients regarding procedures as well as participation in the study. Only the culprit artery was treated, and staged PCI was planned if needed. All PPCI procedures were performed by experienced consultant interventional cardiologists through the radial route. Pre-, peri-, and post-procedure pharmacological therapy and management were uniform for all the patients as per the STEMI guidelines and institutional protocols.

Patient demographics (age and gender) and clinical history of hyperlipidemia, diabetic mellitus, hypertension, and smoking were obtained. Hyperlipidemia was defined as patients with either total cholesterol > 200 mg/dL or triglyceride > 150 mg/dL or low-density lipoprotein > 100 mg/dL or high-density lipoprotein < 40 mg/dL, or on lipid-lowering medications for at least six months. Diabetic mellitus was classified positive for patients with glycosylated hemoglobin (HbA1c) > 6.5% or those taking any oral hypoglycemic medication for a minimum of six months. Similarly, patients on any anti-hypertensive medication for at least six months period were classified as hypertensive. Patients with a history of smoking or currently smoking at least 10 sticks a day for at least one-year duration or equivalent were categorized as smokers. All the included patients were followed during their hospital stay, and outcomes such as mortality and forearm hematoma were recorded. Forearm hematoma of >5 cm in diameter at the radial access site during hospital stay was considered.

All the variables were entered into the Statistical Package for Social Sciences software, version 21 (SPSS Inc., Chicago, IL) for data analysis. Descriptive statistics were computed. Mean ± standard deviation (SD) or median (interquartile range) were computed for quantitative variables, and frequency count with percentage was computed for all categorical variables. The strength of association among different clinical presentations and study outcomes was assessed using the chi-square test, and P-value ≤ 0.05 was considered statistically significant.

## Results

A total of 180 patients with acute STEMI who underwent PPCI through transradial route were included in this study. Age of the patients was in the range of 35 to 75 years, with a mean ± SD of 52.04 ±7.31 years. A majority (87.2%) of patients were male, and diabetes (72.8%) was the most commonly observed co-morbid condition followed by hypertension (67.2%). Co-morbid conditions and demographic distribution of the patients are presented in Table 1.

**Table 1:** Co-morbid conditions and demographic distribution of the patients SD, standard deviation

Clinical Presentation	Frequency	Percentage
Age (mean ± SD), years	52.04±7.31	
≤50 years	85	47.2%
>50 years	98	52.8%
Gender		
Male	157	87.2%
Female	23	12.8%
Co-morbid conditions		
Hypertension	121	67.2%
Diabetic mellitus	131	72.8%
Smoker	82	45.6%
Hyperlipidemia	91	50.6%

In this set of 180 patients, there was access switchover from the transradial route to the transfemoral route, hospital mortality rate was 3.9% (7 patients), and post-procedure forearm hematoma was noted in 5.6% (10 patients) undergoing PCI through the transradial route. An increased mortality rate was found to be associated with age above 50 years (7.1% vs. 0.0%; p=0.012) and non-hypertension (8.5% vs. 1.7%; p=0.026). An increased incidence of forearm hematoma was found to be associated with age above 50 years (10.2% vs. 0.0%; p=0.002), diabetic mellitus (7.6% vs. 0.0%; p=0.047), hyperlipidemia (11% vs. 0.0%; p=0.001), and non-smoking (10.2% vs. 0.0%; p=0.003). In-hospital mortality rate and forearm hematoma by clinical and demographic characteristics are presented in Table 2.

**Table 2:** In-hospital mortality rate and forearm hematoma by clinical and demographic characteristics *Statistically significant at 5%

Clinical Presentation	Base (N)	Mortality Rate	Forearm Hematoma
Age			
≤50 years	85	0% (0)	0% (0)
>50 years	98	7.1% (7)	10.2% (10)
Chi-square test (p-value)	-	0.012*	0.002*
Gender			
Male	157	3.8% (6)	6.4% (10)
Female	23	4.3% (1)	0% (0)
Chi-square test (p-value)	-	0.903	0.213
Hypertension			
Yes	121	1.7% (2)	7.4% (9)
No	59	8.5% (5)	1.7% (1)
Chi-square test (p-value)	-	0.026*	0.114
Diabetic mellitus			
Yes	131	1.5% (2)	7.6% (10)
No	49	10.2% (5)	0% (0)
Chi-square test (p-value)	-	0.433	0.047*
Smoker			
Yes	82	7.3% (6)	0% (0)
No	98	1% (1)	10.2% (10)
Chi-square test (p-value)	-	0.357	0.003*
Hyperlipidemia			
Yes	91	2.2% (2)	11% (10)
No	89	5.6% (5)	0% (0)
Chi-square test (p-value)	-	0.235	0.001*

## Discussion

This study was conducted with an aim to assess the safety of transradial approach for the PPCI in STEMI patients in our population. Hospital mortality rate of 3.9% was noted, post-procedure forearm hematoma was noted in 5.6%, and no procedure needed a crossover or switching access site from the transradial route to the transfemoral route due to any reasons. Cases of forearm hematoma were treatable with conservative measures, did not require surgical strategy, were not life-threatening, and were more common in relatively older patients.

An analysis conducted by Deftereos et al. [21] highlighted that the rate of death was around 11% for those patients who underwent PPCI through a transradial approach. Forearm hematoma was one of the significant problems associated with PPCI using transradial access. Forearm hematoma was observed in 3% of patients who undergone PPCI in one study [21] and was almost 4% in a different analysis [22]. With regard to an elective transradial PCI, a study performed locally showed a really high success rate of 95% [23].

An observational study conducted by Vink et al. [24] established the safety and feasibility of routine use of transradial access for PPCI in patients with STEMI. During the observation period, 96.1% of procedures were performed with transradial access as the primary access. Procedures required crossover of the access sites account for only less than 4% of the total cases, and the trend of crossover continued to decrease with time. In concordance with these findings, no crossover was needed in our study sample.

Periprocedural bleeding is one of the most concerning complications of percutaneous interventions associated with increased mortality and morbidity [25-26]. Various pharmacological options have been tested and recommended for the reduction of bleeding risk; however, in a number of studies, use of a transradial approach was reported to be associated with a reduction in the risk of bleeding [24,27]. Aligned with these findings, post-procedure forearm hematoma was observed in only around 5.6% of the patients in our study and was significantly associated with patients’ baseline characteristics such as increasing age, diabetic mellitus, and hyperlipidemia. It is also observed that the transradial approach is associated with lower contrast volume and fluoroscopy time [28]. Hence, transradial access is safe for PPCI in patients with acute STEMI in our population as hospital mortality rate was only 3.9% and post-procedure forearm hematoma was noted in only 5.6%.

A single-center experience with a limited number of cases and without a comparative group is the key limitation of this study. We also excluded relatively high-risk cases such as patients with cardiogenic shock. Further multicenter and comparative studies will be needed in our population to establish the safety of the transradial approach for PPCI of STEMI patients with varying risk levels and lesion complexities.

## Conclusions

Our study showed that PPCI through a transradial approach is a safe option with excellent success rates in terms of both mortality rates and morbidity such as forearm hematoma. These results were in line with the previous researches, and the transradial approach can be a safe access route for PPCI given sufficient training and exposure of the operator.
